# Ehlers-Danlos Syndrome—Hypermobility Type and Hemorrhoids

**DOI:** 10.1155/2014/171803

**Published:** 2014-04-15

**Authors:** Timothy P. Plackett, Edward Kwon, Ronald A. Gagliano, Robert C. Oh

**Affiliations:** ^1^Department of Surgery, Womack Army Medical Center, 2817 Reilly Road, Fort Bragg, NC 28307, USA; ^2^Landstuhl Regional Medical Center, Geb. 3765, 66849 Landstuhl, Germany; ^3^Tripler Army Medical Center, 1 Jarrett White Road, TAMC, HI 96859, USA; ^4^Fort Belvoir Community Hospital, 9300 DeWitt Loop, Fort Belvoir, VA 22060, USA

## Abstract

Ehlers-Danlos syndrome-hypermobility type (EDS-HT) is a connective tissue disorder associated with chronic musculoskeletal pain. The diagnosis is based on simple clinical examination, although it is easily overlooked. Herein we present a case of EDS-HT associated with hemorrhoids and suggest that there may be an association between the two conditions.

## 1. Introduction

Ehlers-Danlos syndrome (EDS) is a heterogeneous group of connective tissue disorders caused by a deficiency in collagen synthesis and processing. It has been described for centuries and more recently has undergone a change in the classification system. In 1998 the Villefranche nosology grouped the disorder into six broad categories: hypermobility, classical, vascular, kyphoscoliosis, arthrochalasis, and dermatosparaxis [1]. The hypermobility type is second most common variant of EDS and can be associated with cardiovascular and gastrointestinal manifestations [2, 3]. Although hemorrhoids have not been thought of as one of the traditional gastrointestinal manifestations, we present a case that suggests it should be considered as one.

## 2. Case Presentation

A 34-year-old obese female was referred to general surgery for chronic bleeding hemorrhoids. The patient reported a history of intermittent rectal pain, chronic constipation, and occasionally hematochezia. Her physical examination was remarkable for grade II internal hemorrhoids and pelvic floor physiology testing showing an increased sphincter tone.

Additionally, the patient endorsed a history of joint hypermobility, easily bruising, hyperextensive skin, chronic shoulder and elbow dislocations, and chronic back pain. Physical examination of the musculoskeletal system was notable for hyperextension of her knees and elbows, passive apposition of her thumb to forearm, passive hyperextension of her fingers bilaterally, and an ability to place her palms on the floor with forward flexion of her trunk.

Given her symptoms the patient was started on oral stool softeners and fiber supplementation for her hemorrhoids. Surgical intervention of the hemorrhoids was not recommended given the concern for a connective tissue disorder. The patient was referred to rheumatology for further evaluation.

Rheumatology found the patient to meet the criteria for both benign joint hypermobility syndrome and EDS-HT. A subsequent genetic elevation demonstrated similar features in her children but none in prior generations. An echocardiogram was obtained and demonstrated no valvular dysfunction. She was referred to physical therapy to help prevent further joint subluxations.

## 3. Discussion

This is a case of late diagnosis of EDS-HT in a patient who displayed signs of the condition throughout her life. The diagnosis of benign joint hypermobility syndrome and EDS-HT was made through physical examination. Although both conditions are uncommon with an incidence of 1 in 5,000–20,000 individuals, the diseases manifestations can be seen in multiple different manners [4]. For this reason the diagnostic criteria should be familiar to all health care providers (Table 1).

Multiple additional conditions not included in the diagnostic criteria have also been described with EDS. Amongst the more common conditions associated with EDS-HT are Arnold-Chiari type I malformation, dysphonia, and dolichocolon [5, 6]. An association between EDS and anorectal disease has also been previously described. In particular there is an association between EDS and rectal prolapse which has been described in a few small case series and case reports [3]. We believe that hemorrhoids may be another associated condition with EDS. A recent manuscript by Willis and colleagues demonstrated hemorrhoids to be a collagen-based disease, which indirectly supports this hypothesis [7].

The exact mechanism through which EDS-HT is associated with hemorrhoids is not fully elucidated at this time. The genetics for most variants of EDS is fairly well understood, but with EDS-HT it has been more elusive. Mutations of COL5A1 have been found in some, but not all, patients. Mutations of this gene are known to effect type V collagen, which accounts for muscular and tendon stiffness, but no necessarily for the extra-muscular symptoms. Type V collagen is known to influence the formation and overall matrix structure of type I collagen [8]. As hemorrhoid development is associated with decreased numbers of collagen fibers and lower ratio of type I/III collagen fibers, this effect may account for the proposed association between EDS-HT and hemorrhoids [7]. However, this is currently speculative and further study is needed for definitively making this conclusion.

Regardless of whether hemorrhoids are associated with EDS-HT, the presence of EDS requires careful consideration before proceeding with surgical hemorrhoidectomy. EDS patients have a general vascular fragility, which is more prominent is the vascular subtype, but can be seen with other variants [3, 6]. As a result, patients are at higher risk for postoperative bleeding. A single institution study looking at complications after hemorrhoidectomy found that the only patient to require multiple interventions postoperatively to control bleeding was eventually diagnosed with EDS [9]. While treatment needs to be tailored to the individual patient, this report does suggest that when considering surgical intervention the risk of bleeding should be considered higher than traditionally associated with the procedure. In some individuals this risk may outweigh the benefit, especially for less severe hemorrhoid disease.

## 4. Conclusion

In conclusion, the molecular underpinnings of EDS may put the patient at higher risk for hemorrhoidal disease; however the potential for this to be an incidental findings cannot be excluded until further studies are completed. In the meantime, the surgeon evaluating a patient for hemorrhoids should be aware of this potential relationship and if the history is suggestive of concomitant diagnoses appropriate caution should be given before proceeding to a surgical intervention.

## Figures and Tables

**Table 1:** Criteria for diagnosis of Ehlers-Danlos syndrome—hypermobility type.

Major criteria	
(1) Beighton score ≥4	
(2) Arthralgia for greater than 3 months in 4 or more joints	
Minor criteria	
(1) Beighton score 1–3	
(2) Arthralgia in 1–3 joints	
(3) Dislocation of more than 1 joint, or one joint on multiple occasions	
(4) Three or more soft tissue lesions	
(5) Marfanoid body habitus	
(6) Skin striae, hyperextensible skin, thin skin, or abnormal scarring	
(7) Drooping eyes, myopia, or antimongoloid slant	
(8) Varicose veins, hernia, or uterine/rectal prolapse	
